# Effects of the “FIFA11+ Kids” Program on Injury Prevention in Children: A Systematic Review and Meta-Analysis

**DOI:** 10.3390/ijerph191912044

**Published:** 2022-09-23

**Authors:** Jinfeng Yang, Yang Wang, Jianxin Chen, Jinqi Yang, Na Li, Chun Wang, Yuanpeng Liao

**Affiliations:** 1 Department of Sports Medicine and Health, Chengdu Sport University, Chengdu 610041, China; 2 Institute of Sports Medicine and Health, Chengdu Sport University, Chengdu 610041, China; 3 National Clinical Research Center for Geriatrics, West China Hospital of Sichuan University, Chengdu 610041, China; 4 Affiliated Hospital of Chengdu Sport University, Chengdu Sport University, Chengdu 610041, China

**Keywords:** FIFA11+ Kids, football injury, young player, meta-analysis

## Abstract

FIFA11+ Kids is a warm-up program specially designed to prevent football injuries in children. This systematic review and meta-analysis aimed to summarize the effects of FIFA11+ Kids on injury prevention in young football players. PubMed, Web of Science, Embase, Cochrane Library, and China National Knowledge Infrastructure were searched from 1 January 2016 to 24 August 2022. The primary outcome was overall injuries, and the secondary outcomes were severe, ankle, knee, and lower extremity injuries. Risk ratios (RRs) were calculated for each outcome. Methodological quality was assessed using the Physiotherapy Evidence Database (PEDro) scale. A total of 230 articles were screened, 6 of which were finally included in the meta-analysis. Compared with normal warm-up training, FIFA11+ Kids significantly reduced overall injury risk (RR = 0.52 [95% CI, 0.44–0.62]; *p* < 0.00001), severe injury risk (RR = 0.33 [95% CI, 0.18–0.61]; *p* = 0.0004), lower extremity injury risk (RR = 0.51 [95% CI, 0.41–0.65]; *p* < 0.00001), knee injury risk (RR = 0.45 [95% CI, 0.29–0.72]; *p* = 0.0009), and ankle injury risk (RR = 0.56 [95% CI, 0.35–0.89]; *p* = 0.01) in young football players. FIFA11+ Kids was found to be an effective approach to decrease the injury risk among young football players, which is worth generalizing extensively.

## 1. Introduction

As a popular sport worldwide, football attracts more than 400 million people every year [1]. Most people start playing football in childhood [2]. A meta-analysis by Milanović et al. [3] showed that football has a positive impact on physical health, which is reflected in the improvement of cardiovascular, metabolic, and musculoskeletal health, including blood pressure, resting heart rate, fat mass, low-density lipoprotein cholesterol levels, and countermovement jump, and men and women of all ages can experience this positive effect. However, the benefits of football are also accompanied by the occurrence of injuries [4,5], and a large number of minor football players are prone to football injuries, which seriously affects the health and sports performance of minors [6]. Data from the US Emergency Department (ED) show that during the 13 years from 2001 to 2013, there were an estimated 15,960,113 sports-related injuries resulting in ED visits for children aged 5 to 18, with 74.69% of the national estimate coming from football, basketball, American football, and baseball; football is the sport with the most visits, with an estimated number of 4,520,245 [7]. Malina et al. [8] discovered that football players under the age of 14 are at the highest risk of injury among all age groups. Rossler et al. [9] confirmed the view of Malina et al. by following football players aged 7 to 12 for two seasons in the Czech Republic and Switzerland. The injury characteristics of child football players are different from those of football players of other age groups. For example, 60–90% of children’s sports injuries are located in the lower limbs, of which the ankle joint, knee joint, and thigh are the most frequent areas [6]. In addition, the most common types of sports injuries in children are strains (muscle–tendon injuries), sprains (joint–ligament injuries), and contusions [6]. Accordingly, research on football injury prevention among young players is essential.

FIFA11+ is a comprehensive warm-up exercise program designed to reduce injuries among young football players, which was jointly developed in 2006 by the FIFA Medical Assessment and Research Center (Switzerland), the Oslo Sports Trauma Research Center (Norway), and the Saint Monica Sports Medicine Foundation (USA) [10,11]. A study by Soligard et al. [12] showed that FIFA11+ can reduce the risk of injury in young female football players, mainly the risk of serious injury, overuse injury, and overall injury. FIFA11+ was proven to significantly reduce injury rates and decrease days missed because of injury in the competitive male collegiate soccer league [13]. In addition to trials, a meta-analysis by Thorborg et al. [14] provided further evidence that FIFA11+ has a significant injury-reducing effect, reducing football injuries by 39%. However, FIFA11+ is an injury prevention program designed for athletes aged ≥ 14 years, and children who play football lack their proprietary injury prevention program. In 2016, FIFA11+ Kids was developed by F-MARC and related international experts based on FIFA11+ [15]. In particular, FIFA11+ Kids is an injury prevention program specifically designed for children under the age of 14. It lasts 15 min and consists of seven different exercises: a running game, two jumping exercises, a balance/coordination task, two exercises targeting body stability, and an exercise to improve falling technique. These exercises aim to decrease sports injuries among child football players by improving (1) spatial orientation, anticipation, and attention, particularly while dual-tasking (to avoid unintended contact with other players or objects); (2) body stability and movement coordination (more general than specific neuromuscular or proprioceptive training); and (3) learning appropriate fall techniques (to minimize the consequences of unavoidable falls) [15].

Since its inception, FIFA11+ Kids has been introduced to young football players worldwide. When FIFA11+ Kids was developed, it was not found to reduce the incidence of injuries in children participating in football, but to improve children’s sports performance. Rossler et al.—the inventors of FIFA11+ Kids—found that FIFA11+ Kids can improve the sports performance of children aged 7–12 involved in football, mainly by improving dynamic balance and agility and slightly improving jumping performance and slalom dribbling, so they hypothesized that slight improvements in motor performance may reduce the injury risk [15]. Emerging research since then has focused on the effectiveness of FIFA11+ Kids in preventing injuries. Further research by Rossler et al. [16] revealed that FIFA11+ Kids is effective in reducing injuries in young football players; the overall injury rate in the intervention group was reduced by 48% compared with the control group (hazard ratio 0.52; 95% confidence interval [CI], 0.32–0.86). Severe (74% reduction, hazard ratio 0.26; 95% CI 0.10–0.64) and lower extremity injuries (55% reduction, hazard ratio 0.45; 95% CI 0.24–0.84) were also reduced. In addition, Zarei et al. [17] demonstrated that with FIFA11+ Kids, the overall incidence of injuries per 1000 h of football exposure was reduced by 50% compared with normal warm-up among high-level young football players (rate ratio 0.50; 95% CI 0.32–0.78). However, as a novel injury prevention training, robust evidence from high standard systematic reviews and meta-analyses is still lacking. Therefore, the purpose of this systematic review and meta-analysis was to synthesize and assess the effects of FIFA11+ Kids on injury prevention in children in order to further refine the guidelines for children’s football injury prevention.

## 2. Materials and Methods

This systematic review and meta-analysis followed the guidelines provided in the Preferred Reporting Items for Systematic Reviews and Meta-Analyses (PRISMA) statement, which is a 27-item checklist of all steps [18]. The study protocol has been registered in PROSPERO under registration number CRD42021283043.

### 2.1. Eligibility Criteria

#### 2.1.1. Inclusion Criteria

Inclusion criteria for all studies were based on the population, intervention, comparison, outcomes, and study design (PICOS) principle [18]. Studies were included for meta-analysis if they met the following criteria: (1) subjects were child football players; (2) the intervention program was FIFA11+ Kids, which compared with normal warm-up training, both with a clear training frequency and duration; (3) the primary outcome was the total number of injuries of each type in football, and additional outcomes were the number of severe injuries and injuries to different body regions in football; (4) the types of studies were randomized controlled trials (RCTs) or cluster-randomized trials; (5) full-text papers were available.

#### 2.1.2. Exclusion Criteria

The exclusion criteria were as follows: (1) studies that had other interventions, such as tape and drug interventions; (2) studies not published in Chinese or English; (3) case reports, systematic reviews, interviews, and conference abstracts; and studies with quasi-random allocation, without available data, and duplicate publications.

### 2.2. Information Sources

FIFA11+ Kids is a novel injury prevention program which was developed in 2016 [15]; thus, we excluded literature before 2016 when searching. Two reviewers (J.Y. (Jinfeng Yang) and J.C.) independently searched the following electronic databases for relevant studies published from January 2016 (when FIFA11+ Kids was launched) to August 2022: PubMed, Web of Science, Embase, Cochrane Library, and China National Knowledge Infrastructure. In addition, the reference lists of eligible studies were searched.

### 2.3. Search Strategy

Search terms were combined by Boolean logic (AND [between categories], OR [within categories]). The search strategy of all databases is given in Table A1. There were no study design limits and only studies published in Chinese or English were included.

### 2.4. Selection Process

The retrieved studies were first imported into Endnote software (Version X9; Thomson Reuters) for repetition processing. According to the established inclusion and exclusion criteria, two reviewers (J.Y. (Jinqi Yang) and C.W.) further reviewed studies for the content of titles and abstracts and read the full text of studies that met the criteria to determine the final inclusion. After screening, the results from the two reviewers were compared. Disagreements were resolved through discussion with a third reviewer (N.L.).

### 2.5. Data Collection Process

The two reviewers (J.Y. (Jinfeng Yang) and J.C.) extracted the data of interest from the selected studies using a self-designed data acquisition form in Excel (Version 2013; Microsoft, Redmond, WA, USA). Disagreements were resolved by discussion or consultation with a third reviewer (N.L.). If study data were lacking or unclear, then the reviewer (Y.W.) would contact the author through various methods to obtain complete information. Studies without available data were excluded.

### 2.6. Data Items

The extracted data included (1) basic information on the studies, such as the author of the studies and the publication period, and (2) experimental characteristics of the studies, such as sample size, age, intervention measures, intervention frequency, intervention period, and outcome indicators. According to a highly cited consensus statement, the injury was defined as “any physical complaint sustained by a player that results from a football match or football training”, and the severe injury was defined as “any injury that leading to an absence longer than 28 days [19]. The primary outcome was overall injuries defined as the number of all injuries during the study period in football play. The secondary outcomes were severe, ankle, knee, and lower extremity injuries, all defined as the number of injuries during the study period in football play, respectively.

### 2.7. Study Risk of Bias Assessment

Two reviewers (J.Y. (Jinqi Yang) and C.W.) used the Physiotherapy Evidence Database (PEDro) scale, which is a rating scale to assess the quality of studies [20]. This assessment was used to assign a value ranging from 0 (high risk of bias) to 10 (low risk of bias) to the included studies, according to 11 items developed from the Delphi list to identify the quality of RCTs [21]. Its items mostly assess factors related to the risk of bias in studies. The total PEDro score was obtained by adding the ratings of items 2 to 11 for a combined total score between 0 and 10 [20]. Scores under 4 were considered as “poor”, 4 to 5 as “fair”, 6 to 8 as “good”, and 9 to 10 as “excellent”. If the results of the methodological quality assessment from the two reviewers were inconsistent, then the third reviewer (Y.W.) was consulted to resolve such inconsistencies. The 11 items include (1) eligibility criteria and source, (2) random allocation, (3) concealed allocation, (4) baseline comparability, (5) blinding of participants, (6) blinding of therapists, (7) blinding of assessors, (8) adequate follow-up (>85%), (9) intention-to-treat analysis, (10) between-group statistical comparisons, and (11) reporting of point estimates and variability.

### 2.8. Effect Measures

The outcome variables in this meta-analysis were dichotomous, so we chose risk ratio (RR) with 95%CI to express the pooled results, at a significance level of *p* < 0.05 [22]. If RR < 1, the intervention had a positive effect on reducing the incidence of injury. The lower the RR value, the more significant the reduction effect.

### 2.9. Synthesis Methods

All cluster-randomized trials were adjusted for cluster effects in all pooled analyses. We used the intracluster correlation coefficient (ICC) from a similar previous study (ICC = 0.05) [12] to adjust for potential cluster effects by calculating the inflation factor (IF) [22]. The IF was calculated as follows: IF = 1 + (n − 1)ρ, where ρ is the ICC and *n* is the average cluster size [23]. The effective sample size was calculated by dividing the number of injuries and participants by IF [22]. The forest plots and the I-squared statistics were used to examine the variability and heterogeneity across studies. I^2^ < 25% indicated low heterogeneity among the studies; 25% ≤ I^2^ < 50% indicated moderate heterogeneity among the studies, and I^2^ ≥ 50% indicated high heterogeneity among the studies [24]. The Mantel-Haenszel random-effects method was selected for all analyses. All analyses were run in Review Manager software (Version 5.3; Cochrane Collaboration) by one reviewer (J.Y. (Jinfeng Yang)).

### 2.10. Reporting Bias Assessment

Since the number of included studies was only six, reporting bias analysis was omitted.

### 2.11. Certainty Assessment

In addition, two reviewers (J.Y. (Jinfeng Yang) and J.C.) used GRADE profiler software (Version 3.6; Cochrane IMS) to assess the quality of evidence [25]. Based on the results of the systematic review, the quality of the evidence was assessed using the GRADE recommendation grading method. The quality of the evidence was graded as follows: (1) high quality—further research is unlikely to change the credibility of the efficacy assessment results; (2) moderate quality—further research is likely to affect the credibility of the assessment results and may change the assessment results; (3) low quality—further research is very likely to affect the credibility of the efficacy assessment results and the assessment results are likely to change; (4) very low quality—the results of any efficacy assessment are very uncertain. In the event of discrepancies, a third reviewer (N.L.) was involved.

## 3. Results

### 3.1. Study Selection

**Figure 1:**
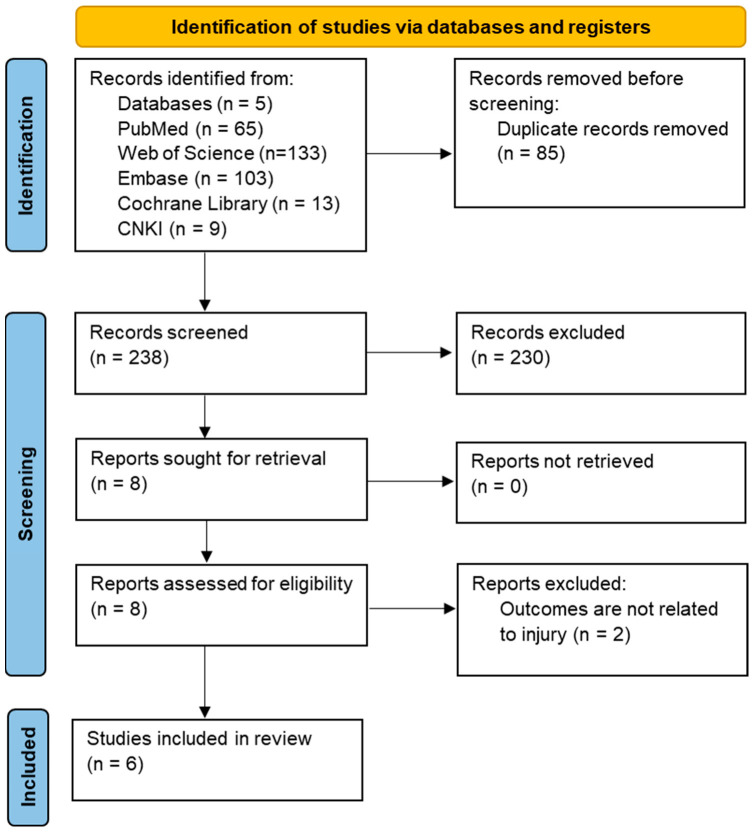
Screening process for systematic review in accordance with PRISMA.

A total of 323 studies were initially retrieved from various databases. After excluding 85 duplicate studies, 238 studies were obtained. Then, the titles and abstracts were read, following which 8 studies were selected. Of these, 2 studies were excluded because of unrelated outcomes [15,26]. Finally, 6 studies were included in the review [16,17,27,28,29,30]. The screening of specific studies was in accordance with the PRISMA guidelines. The total selection process is shown in Figure 1.

### 3.2. Study Characteristics

One of the six studies was an RCT [29], and the other five were cluster-randomized trials [16,17,27,28,30]. A total of 10,565 participants were included in this analysis, all of which were football players aged 7–14 years. The intervention method of these six studies was FIFA11+ Kids, the intervention frequency was two to three times per week, and the intervention period was 3–10 months. Normal warm-up training was used for comparison in all six studies. Outcome indicators included the number of overall, severe, lower extremity, knee, and ankle injuries. Of the six included studies, three were multicenter studies in four countries (Switzerland, Germany, Czech Republic, and The Netherlands) [16,27,28], and the remaining three studies were conducted in Saudi Arabia [30], China [29], and Iran [17]. Two of the studies only included boys [17,30], and the other four studies included both boys and girls [16,27,28,29]. The participants in four of these studies were young football players from officially registered football clubs [16,27,28,30], whereas the subjects in the other two studies were football players affiliated with male youth football teams that compete in the highest league of Iranian provinces [17] and fourth-grade students in China [29], respectively. Table A2 summarizes the characteristics of the six included studies.

### 3.3. Risk of Bias in Studies

The methodological quality of the six included studies was “good”. Four studies’ PEDro scores were 6 [16,17,27,29] and two studies were rated 7 [28,30]. These PEDro scores indicate a low risk of bias in the six included studies (Table A3).

### 3.4. Effect of FIFA11+ Kids on Injury among Young Football Players

#### 3.4.1. Overall Injury

**Figure 2:**
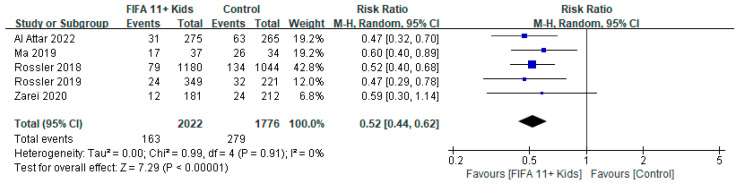
Primary analysis of overall injury rates in the “FIFA11+ Kids” program compared with control intervention on overall number of injuries [16,17,27,29,30].

With respect to the primary outcome of overall injury, meta-analysis of the FIFA11+ Kids and control groups in five studies [16,17,27,29,30] revealed the following: RR = 0.52, 95% CI (0.44, 0.62), *p* < 0.00001, I^2^ = 0%. These results indicated a significant reduction in the risk of overall injury in the FIFA11+ Kids group compared with the control group (Figure 2).

#### 3.4.2. Injury Severity

**Figure 3:**
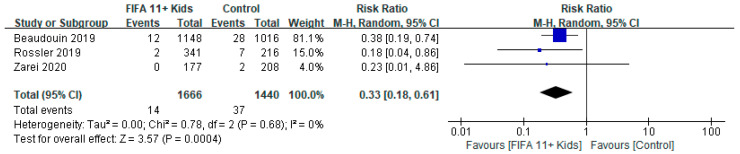
Secondary analysis of severe injury rates in the “FIFA11+ Kids” program compared with control intervention on the number of severe injuries [17,27,28].

With respect to the secondary outcome of injury severity, meta-analysis of the FIFA11+ Kids and control groups in four studies [17,27,28] revealed the following: RR = 0.33, 95% CI (0.18, 0.61), *p* = 0.0004, I^2^ = 0%. These results indicate a significant reduction in the risk of severe injury in the FIFA11+ Kids group compared with the control group (Figure 3).

#### 3.4.3. Injury in Different Body Regions

**Figure 4:**
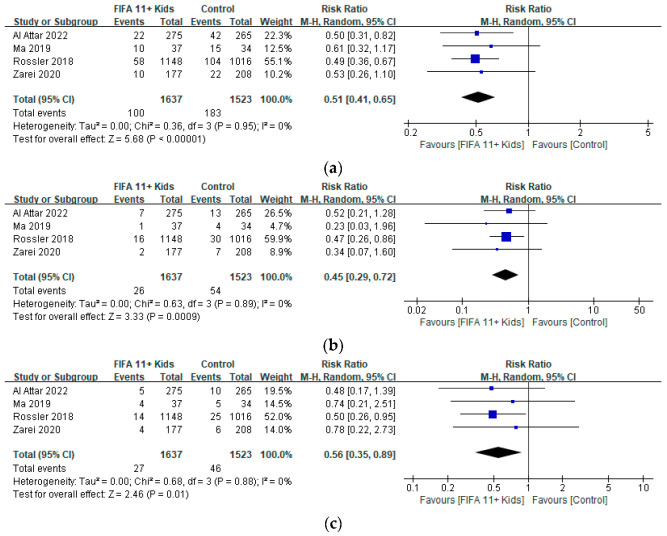
(**a**) Secondary analyses of injury rates in relation to conducting the “FIFA11+ Kids” Program compared with control intervention on the number of lower extremity injuries [16,17,29,30]. (**b**) Secondary analyses of injury rates in relation to conducting the “FIFA11+ Kids” Program compared with control intervention on the number of knee injuries [16,17,29,30]. (**c**) Secondary analyses of injury rates in relation to conducting the “FIFA11+ Kids” Program compared with control intervention on the number of ankle injuries [16,17,29,30].

With respect to the secondary outcome of the injured body region, meta-analysis of the incidence of lower extremity injuries in the FIFA11+ Kids and control groups in four studies [16,17,29,30] revealed the following: RR = 0.51, 95% CI (0.41, 0.65), *p* < 0.00001, I^2^ = 0%. These results indicate a significant reduction in the risk of lower extremity injury in the FIFA11+ Kids group compared with the control group (Figure 4a). Meta-analysis of the incidence of knee injuries in the FIFA11+ Kids and control groups in four studies [16,17,29,30] revealed the following: RR = 0.45, 95% CI (0.29, 0.72), *p =* 0.0009, I^2^ = 0%. These results indicate a significant reduction in the risk of knee injury in the FIFA11+ Kids group compared with the control group (Figure 4b). Meta-analysis of the incidence of ankle injuries in the FIFA11+ Kids and control groups in four studies [16,17,29,30] revealed the following: RR = 0.56, 95% CI (0.35, 0.89), *p* = 0.01, I^2^ = 0%. These results indicate a significant reduction in the risk of ankle injury in the FIFA11+ Kids group compared with the control group (Figure 4c).

### 3.5. Certainty of Evidence

The results of the GRADE system were expressed in the format of “Summary of Findings Table,” and the outcome indicators included overall injury, lower extremity injury, knee injury, ankle injury, and severe injury. The quality of evidence was moderate for the first four outcomes and low for the last serious injury (Table A4).

## 4. Discussion

An increasing number of children have been involved in sports in recent years [31]; as such, the problem of child injuries in football has attracted increasing attention. Therefore, a comprehensive football injury prevention program is essential for children involved in football. Although a systematic review of FIFA11+ for injury prevention and performance improvement has been published this year, it was still evaluating the effect of FIFA11+ on football players of all ages [32]. No robust evidence from high-quality meta-analyses to evaluate the preventive effect of FIFA11+ Kids on football injury in children has been published. Therefore, we evaluated the preventive effects of FIFA11+ Kids on injury among young football players. We included six studies with 10,565 participants, and the results showed that FIFA11+ Kids was more effective than regular warm-up training in reducing injury rates in young football players.

The methodological quality of these included studies was “good”, and the risk of bias was manageable. However, the quality of the evidence was not good enough. The quality of evidence was moderate for four outcomes (overall, lower extremity, knee, and ankle injuries) and low for one outcome (severe injury), mainly because of the lack of blinding of participants and personnel. Previous systematic reviews have shown that blinding is the most common reason for the quality of evidence in physical exercise studies [14,33]. Indeed, blinding is difficult in similar studies if the investigator is involved in the training. Thus, we suggest that the investigator as well as the participants be blinded in future studies. Code names can be assigned to different groups, where neither the investigator nor the participants know the allocation. In this way, double blinding could be realized in physical exercise studies, and the quality of evidence can be improved.

Three of the included studies in this review were from European countries [16,27,28], which may be because Europe is the continent with the most developed football youth training and the highest football penetration rate in children (event rate: 28.5%; 95% CI 20.9% to 37.7%) [34]. The remaining three studies were from Saudi Arabia [30], China [29] and Iran [17], with significantly smaller sample sizes than the studies conducted in Europe. This is because the football participation rate of children in the Western Pacific is only the sixth highest among all sports (event rate: 18.1%; 95% CI 7.1% to 39.1%) [34]. Of the six included studies, two studies had only male participants [17,30], and the remaining four included both male and female participants [16,27,28,29]. The studies with only male participants were from Saudi Arabia [30] and Iran [17], where exercise among females may be affected by the religious culture. The remaining four studies all included female participants, with three studies having a low proportion of females (4.4–7.4%) [16,27,28], suggesting that football is far less popular among girls than among boys. We should encourage girls to play football and enjoy the same benefits of football as boys. The intervention periods of FIFA11+ Kids are various in the included studies. Three studies have an intervention period of 10 weeks [16,27,28], one has an intervention period of 9 weeks [17], one has an intervention period of 6 weeks [30], and the last has a shorter period of only 3 weeks [29]. All studies showed reduced injury rates in children participating in football, suggesting that FIFA11+ Kids has a significant preventive effect on injury even with short-term intervention. The included studies also demonstrated that FIFA11+ Kids had a significant preventive effect on football injury in children at the elite, sub-elite, and amateur levels, reflecting the universality of the program.

In this systematic review and meta-analysis, the primary outcome was the overall incidence of injury. The main finding was that FIFA11+ Kids significantly decreased the risk of overall injury compared with normal warm-up training. Football players under the age of 14 are at the highest risk of injury among all age groups [8]. Our meta-analysis demonstrated the effectiveness of FIFA11+ Kids in preventing football-related injury in young players. Previous research has shown that serious injury cases are more than a quarter of all injuries, and severe injuries seriously affect the training of athletes [35]. In particular, the incidence of fractures in children under the age of 15 is higher than that in adults, and a certain correlation may be found between the level of physical development and the characteristics of sports injuries [10]. Based on our secondary analyses, we found that FIFA11+ Kids is designed for preventing not only overall injury but also severe injury. Fractures are the most common severe injury in football. Bizzini et al. [10] believed that the incidence of fractures in children under the age of 15 is higher than that in adults because different levels of physical development lead to various characteristics of sports injuries. Soligard et al. [12] found that inadequate coordination may influence the risk of fractures in children. Whether FIFA11+ Kids can improve the physical development level remains debatable, but FIFA11+ Kids can improve body stability and movement coordination [36], which might be the key mechanisms through which FIFA11+ Kids prevent severe injury.

In our secondary analyses of the injured body region, results showed that FIFA11+ Kids could significantly decrease the risk of ankle, knee, and lower extremity injuries. Lower extremity injuries are common among football players, with thigh strains accounting for the highest proportion [37]. In particular, among professional male youth football players, the number of lower extremity injuries has been stable [38]. Read et al. [39] found that single-leg countermovement jump peak landing force asymmetry is the main risk factor for lower extremity injury. We hypothesize that FIFA11+ Kids can play a good preventive role by changing landing force asymmetry because the neuromuscular control ability is a modifiable risk factor [40,41]. Ankle and knee injuries are the most common types of injuries. For example, the frequency of rapid directional changes and high-intensity jump landing events that occur during exercise leads to increased rates of medial collateral ligament injuries [42]. Video analysis has shown that the lateral force in direct contact with the inner calf before or when the foot hits the ground, causing players to land on their ankles in this vulnerable inverted position, is a major cause of ankle injuries in football players [43]. Many studies have proven that FIFA11+ Kids can effectively improve sports performance among children [15,36,44,45,46]. Zarei et al. [26] also found that FIFA11+ Kids showed significant improvements in isokinetic strength of the hip adductors, knee flexors, ankle evertors, and ankle invertors compared with usual warm-up training. These positive effects result in a stronger body with better handling techniques for contact situations and higher load tolerance, thereby reducing the risk of injury to the lower extremities, particularly the knee [47]. This is a good indicator of further research on the injury preventive effects of FIFA11+ Kids on different body regions among young football players.

The present study is the first systematic review of FIFA11+ Kids, which has some limitations in common with previous systematic reviews of FIFA11+. Firstly, blinding of participants and therapists in FIFA11+ Kids is difficult, which affects the methodological quality of the studies. We propose that future studies should achieve double blinding of physical exercise studies to enhance the quality of studies by using various methods such as assigning code names to different groups. Secondly, while most current studies included both boys and girls, the proportion of girls was very low. Therefore, future studies can focus on whether there are gender differences in the injury preventive effect of FIFA11+ Kids. In addition, there are regional differences in the FIFA11+ Kids studies similar to the FIFA11+ studies, and the FIFA11+ Kids studies included in this systematic review were mainly conducted in Europe. Future research should focus on the effects of FIFA11+ Kids on other continents and children of different ethnicities. Finally, because we only included studies published in Chinese or English, there are some language restrictions on the search strategy of this systematic review. We suggest that future systematic reviews use more diverse languages for study search and inclusion.

It should also be noted that FIFA11+ Kids is a novel football injury prevention strategy for children that was proposed less than 10 years ago [15], and related research is limited. Therefore, the available clinical evidence for this study was sparse, and only six FIFA11+ Kids studies were included in our systematic review, which was much less than the number of studies included in systematic reviews of FIFA11+ [33,48]. In the future, concerns about injury prevention in young football players should be raised by researchers. A series of high-quality RCTs exploring the effects of different intervention frequencies, durations, and intensities of FIFA11+ Kids on young football players are needed to further illustrate our research conclusions.

## 5. Conclusions

This meta-analysis provided positive evidence regarding the preventive effects of FIFA11+ Kids on injury in young football players. In addition to the risk of overall injury, the risks of severe, lower extremity, knee, and ankle injuries could be reduced by FIFA11+ Kids; this suggests that FIFA11+ Kids should be extensively promoted. Future research can provide interventions for different groups with different code names to improve research quality.

## Data Availability

Not applicable.

## Appendix A

**Table A1:** Search strategies for all databases.

(a) PubMed	
No.	Search Details
#1	“Football”[MeSH] OR “Soccer”[MeSH] OR “Football”[Title/Abstract] OR “Soccer” [Title/Abstract]
#2	“Warm-Up Exercise”[MeSH] OR “FIFA11+ Kids”[Title/Abstract] OR “11+ Kids”[Title/Abstract] OR “Warm-Up Exercise*”[Title/Abstract] OR “Warm-Up Program*”[Title/Abstract] OR “Injury Prevention”[Title/Abstract]
#3	“Child”[MeSH] OR “Child”[Title/Abstract] OR “Children”[Title/Abstract]
#4	“Athletic Injuries”[MeSH] OR “Athletic Injur*”[Title/Abstract] OR “Sports Injur*”[Title/Abstract] OR “Injury Rate*”[Title/Abstract]
#5	#1 AND #2 AND #3 AND #4
Timespan = 2016–2022	
**(b) Web of Science**	
**No.**	**Search Details**
#1	TS = (‘Football’ OR ‘Soccer’)
#2	TS = (‘FIFA11+ Kids’ OR ‘11+ Kids’ OR ‘Warm-Up Exercise*’ OR ‘Warm-Up Program*’ OR ‘Injury Prevention’)
#3	TS = (‘Child’ OR ‘Children’)
#4	TS = (‘Athletic Injur*’ OR ‘Sports Injur*’ OR ’Injury Rate*’)
#5	#1 AND #2 AND #3 AND #4
Timespan = 2016–2022	
Databases = SCI-EXPANDED, SSCI, A&HCI, CPCI-S, CPCI-SSH, ESCI	
**(c) Embase**	
**No.**	**Search Details**
#1	‘football’/de OR ‘soccer’/de OR ‘football’:ab,ti OR ‘soccer’:ab,ti
#2	‘warm-up exercise’/de OR ‘FIFA11+ kids’:ab,ti OR ‘11+ kids’:ab,ti OR ‘warm-up exercise*’:ab,ti OR ‘warm-up program*’:ab,ti OR ‘injury prevention’:ab,ti
#3	‘child’/de OR ‘child’:ab,ti OR ‘children’:ab,ti
#4	‘athletic injuries’/de OR ‘athletic injur*’:ab,ti OR ‘sports injur*’:ab,ti OR ‘injury rate*’:ab,ti
#5	#1 AND #2 AND #3 AND #4
Timespan = 2016–2022	
**(d) Cochrane Library**	
**No.**	**Search Details**
#1	MeSH descriptor: [Football] explode all trees
#2	MeSH descriptor: [Soccer] explode all trees
#3	(Football OR Soccer):ti,ab,kw
#4	#1 OR #2 OR #3
#5	MeSH descriptor: [Warm-Up Exercise] explode all trees
#6	(“FIFA11+ Kids” OR “11+ Kids” OR “Warm-Up Exercise*” OR “Warm-Up Program*” OR “Injury Prevention”):ti,ab,kw
#7	#5 OR #6
#8	MeSH descriptor: [Child] explode all trees
#9	(Child OR Children):ti,ab,kw
#10	#8 OR #9
#11	MeSH descriptor: [Athletic Injuries] explode all trees
#12	(“Athletic Injur*” OR “Sports Injur*” OR “Injury Rate*”):ti,ab,kw
#13	#11 OR #12
#14	#4 AND #7 AND #10AND #13
Timespan = 2016–2022	
**(e) China National Knowledge Infrastructure**	
**No.**	**Search Details**
#1	主题 (Topic) = 足球 (Football, Soccer)
#2	主题 (Topic) = FIFA11+ Kids OR The 11+ Kids OR 热身 (Warm-up Exercise) OR 伤病预防 (Injury Prevention)
#3	主题 (Topic)= 儿童 (Child, Children)
#4	主题 (Topic) = 运动损伤 (Athletic Injury) OR 运动伤病 (Sports Injury) OR 受伤 (Injury)
#5	#1 AND #2 AND #3 AND #4
Timespan = 2016–2022	

**Table A2:** Characteristics of the included studies.

Study	Participants	Level	N	Age (Years)	Intervention	Comparison	Frequency (No. Per Week)	Duration	Study Design
Al Attar et al. (2022) [30]	Young football players, Boy	Sub-elite	377	7–13	FIFA11+ Kids	Normal warm-up training	At least 2/week	6 months	cluster RCT
			363						
Beaudouin et al. (2019) [28]	Young football players, Boy and Girl	Sub-elite	2066	11.7 ± 0.8	FIFA11+ Kids	Normal warm-up training	2/week	10 months	cluster RCT
			1829	11.3 ± 1.2					
Ma (2019) [29]	Primary school students, Boy and Girl	Amateur	37	Fourth-grade primary school	FIFA11+ Kids	Normal warm-up training	3/week	3 months	RCT
			34						
Rossler et al. (2018) [16]	Young football players, Boy and Girl	Sub-elite	2066	10.8 ± 1.4	FIFA11+ Kids	Normal warm-up training	2/week	10 months	cluster RCT
			1829	10.7 ± 1.4					
Rossler et al. (2019) [27]	Young football players, Boy and Girl	Sub-elite	614	11.0 ± 1.2	FIFA11+ Kids	Normal warm-up training	2/week	10 months	cluster RCT
			388	10.6 ± 1.1					
Zarei et al. (2020) [17]	Young football player, Boy	Elite	443	12.1 ± 1.8	FIFA11+ Kids	Normal warm-up training	At least 2/week	9 months	cluster RCT
			519	12.2 ± 1.7					

**Table A3:** Study quality on the PEDro scale.

Study	1	2	3	4	5	6	7	8	9	10	11	Total
Al Attar et al. (2022) [30]	1	1	1	1	0	0	0	1	1	1	1	7
Beaudouin et al. (2019) [28]	1	1	0	1	0	0	1	1	1	1	1	7
Ma (2019) [29]	1	1	0	1	0	0	0	1	1	1	1	6
Rossler et al. (2018) [16]	1	1	1	0	0	0	1	1	0	1	1	6
Rossler et al. (2019) [27]	1	1	1	0	0	0	0	1	1	1	1	6
Zarei et al. (2020) [17]	1	1	1	1	0	0	0	0	1	1	1	6

Note: (1) Eligibility criteria and source, (2) random allocation, (3) concealed allocation, (4) baseline comparability, (5) blinding of participants, (6) blinding of therapists, (7) blinding of assessors, (8) adequate follow-up (>85%), (9) intention-to-treat analysis, (10) between-group statistical comparisons, and (11) reporting of point estimates and variability. Item 1 does not contribute to the total score.

**Table A4:** GRADE Profile of the Included Studies.

FIFA11+ Kids Compared to Normal Warm-Up for Children					
**Patient or population:** children **Settings:** FIFA11+ Kids compared to normal warm-up training in injury prevention of children **Intervention:** FIFA11+ Kids **Comparison:** Normal warm-up training					
**Outcomes**	**Illustrative comparative risks * ** **(95% CI)**		**Relative Effect** **(95% CI)**	**No of Participants** **(Studies)**	**Quality of the Evidence** **(GRADE)**
	**Normal warm-up**	**FIFA11+ Kids**			
**Overall Injury** Follow-up: 3–10 months	**158 per 1000**	**84 per 1000** (73 to 98)	**RR 0.53** (0.46 to 0.62)	3797 (5 studies)	⊕⊕⊕⊝ **moderate** ^1^
**Severe Injury** Follow-up: 9–10 months	**25 per 1000**	**8 per 1000** (5 to 13)	**RR 0.32** (0.21 to 0.51)	3645 (4 studies)	⊕⊕⊝⊝ **low** ^1,2^
**Lower Extremity Injury** Follow-up: 3–10 months	**120 per 1000**	**61 per 1000** (50 to 74)	**RR 0.51** (0.42 to 0.62)	3160 (4 studies)	⊕⊕⊕⊝ **moderate** ^1^
**Knee Injury** Follow-up: 3–10 months	**35 per 1000**	**16 per 1000** (11 to 23)	**RR 0.45** (0.3 to 0.66)	3160 (4 studies)	⊕⊕⊕⊝ **moderate** ^1^
**Ankle Injury** Follow-up: 3–10 months	**47 per 1000**	**27 per 1000** (18 to 40)	**RR 0.57** (0.39 to 0.85)	3160 (4 studies)	⊕⊕⊕⊝ **moderate** ^1^

* The basis for the assumed risk (e.g., the median control group risk across studies) is provided in footnotes. The corresponding risk (and its 95% confidence interval) is based on the assumed risk in the comparison group and the relative effect of the intervention (and its 95% CI). CI: Confidence interval; RR: Risk ratio; ^1^ There was no blinding for participants and personnel in some included studies. ^2^ One study was a secondary analysis of data from a multicenter cluster-randomized controlled trial.
